# A new hyperpolarized ^13^C ketone body probe reveals an increase in acetoacetate utilization in the diabetic rat heart

**DOI:** 10.1038/s41598-019-39378-w

**Published:** 2019-04-02

**Authors:** Desiree Abdurrachim, Chern Chiuh Woo, Xing Qi Teo, Wei Xin Chan, George K. Radda, Philip Teck Hock Lee

**Affiliations:** 1 Functional Metabolism Group, Singapore Bioimaging Consortium, Agency for Science, Technology, and Research, Singapore, Singapore; 2 Department of Physiology, Anatomy and Genetics, University of Oxford, Oxford, United Kingdom

## Abstract

Emerging studies have recently shown the potential importance of ketone bodies in cardio-metabolic health. However, techniques to determine myocardial ketone body utilization *in vivo* are lacking. In this work, we developed a novel method to assess myocardial ketone body utilization *in vivo* using hyperpolarized [3-^13^C]acetoacetate and investigated the alterations in myocardial ketone body metabolism in diabetic rats. Within a minute upon injection of [3-^13^C]acetoacetate, the production of [5-^13^C]glutamate and [1-^13^C] acetylcarnitine can be observed real time *in vivo*. In diabetic rats, the production of [5-^13^C]glutamate was elevated compared to controls, while [1-^13^C]acetylcarnitine was not different. This suggests an increase in ketone body utilization in the diabetic heart, with the produced acetyl-CoA channelled into the tricarboxylic acid cycle. This observation was corroborated by an increase activity of succinyl-CoA:3-ketoacid-CoA transferase (SCOT) activity, the rate-limiting enzyme of ketone body utilization, in the diabetic heart. The increased ketone body oxidation in the diabetic hearts correlated with cardiac hypertrophy and dysfunction, suggesting a potential coupling between ketone body metabolism and cardiac function. Hyperpolarized [3-^13^C]acetoacetate is a new probe with potential for non-invasive and real time monitoring of myocardial ketone body oxidation *in vivo*, which offers a powerful tool to follow disease progression or therapeutic interventions.

## Introduction

The mammalian heart is analogous to a multi-fuel engine. While a major proportion of its energy requirement is typically fulfilled by fatty acids and glucose, the heart has the capacity to utilize other organic substrates such as ketone bodies, lactate, and amino acids^1^. Research on cardiac energy metabolism has largely focused on fatty acids and glucose utilization. However, there is a growing interest in ketone body metabolism^2^ as recent studies have shown its importance in cardio-metabolic health. Increased myocardial ketone body utilization has been reported in the hypertrophied and failing mouse hearts^3^ and in the end-stage human heart failure^4^. Interestingly, mice with a cardiac-specific deficiency in succinyl-CoA:3-ketoacid-CoA transferase (SCOT), which is the rate-limiting enzyme involved in the first step of ketone body metabolism, had exacerbated cardiac hypertrophy and dysfunction in response to pressure-overload^5^. In addition, intravenous infusion of β-hydroxybutyrate (β-OHB) together with prolonged fasting has been shown to reduce infarct size by 46% in a rat model of myocardial infarction^6^. These studies indicate the emerging importance of understanding altered ketone body metabolism in cardiac pathophysiology, and suggest that its pharmacological modulation may be a potential therapeutic strategy.

To date, there are limited non-radioactive and non-invasive methods to directly assess myocardial ketone oxidation *in vivo*. Ziegler *et al*. determined the contribution of [2,4-^13^C]β-OHB in the production of acetyl-CoA for incorporation into the tricarboxylic acid (TCA) cycle in the healthy rat heart using *in vivo* ^13^C magnetic resonance spectroscopy (MRS)^7^. However, a long infusion time of 2 hours was required, which limited direct and real time observation. Indirect techniques such as nuclear magnetic resonance (NMR) of tissue extracted from rat heart perfused with ^13^C-labeled isotopic tracers^8,9^, liquid chromatography-mass spectrometry analysis of myocardial biopsies^4^, or sampling of circulating ketone bodies^10^ have also been investigated. Although radioactive ketone tracers (i.e. ^11^C-β-OHB and ^11^C-acetoacetate) have been used to measure ketone metabolism in the brain^11,12^ and in the heart^13^, these PET agents do not directly reveal their metabolic fates or enzyme activity.

The advent of hyperpolarized ^13^C MRS has revolutionized the *in vivo* metabolic imaging landscape^14^. Not only does this technology permit real-time detection of specific metabolic pathways^15,16^, enzyme kinetics can be modelled accurately because the downstream metabolites are distinctly captured in the chemical shift domain^17^. The potential application of hyperpolarized ^13^C MRS for measuring ketone metabolism *in vivo* was recently highlighted in a study of short-chain fatty acid metabolism of ^13^C-butyrate^18^. However, the detected ^13^C-acetoacetate metabolite was the product derived from the SCOT-mediated conversion of acetoacetyl-CoA, which itself was an intermediate in the β-oxidation of butyrate. Recently, hyperpolarized [1-^13^C]β-OHB^19^, [1-^13^C]acetoacetate^19^, and [1,3-^13^C_2_]acetoacetate^20^, which provide more direct information on ketone body oxidation, have been reported.

In this work, we synthesized lithium [3-^13^C]acetotacetate for hyperpolarization and demonstrated the use of the probe to measure myocardial ketone body utilization *in vivo* in Goto-Kakizaki rats, a non-obese model of type 2 diabetes mellitus^21^. We observed that the ^13^C label incorporation from [3-^13^C]acetoacetate into [5-^13^C]glutamate, a TCA cycle product, was higher in the diabetic heart compared with controls, indicating increased ketone oxidation in the diabetic heart. *Ex vivo* biochemical analysis revealed higher SCOT activity in the diabetic heart, therefore validating the *in vivo* hyperpolarized ^13^C MRS results. Interestingly, higher ketone body utilization in the diabetic heart was shown to correlate with lower ejection fraction in the diabetic heart, which demonstrates potentially a tight coupling between ketone body metabolism and cardiac function.

## Results

### Animal characteristics

**Figure 1:**
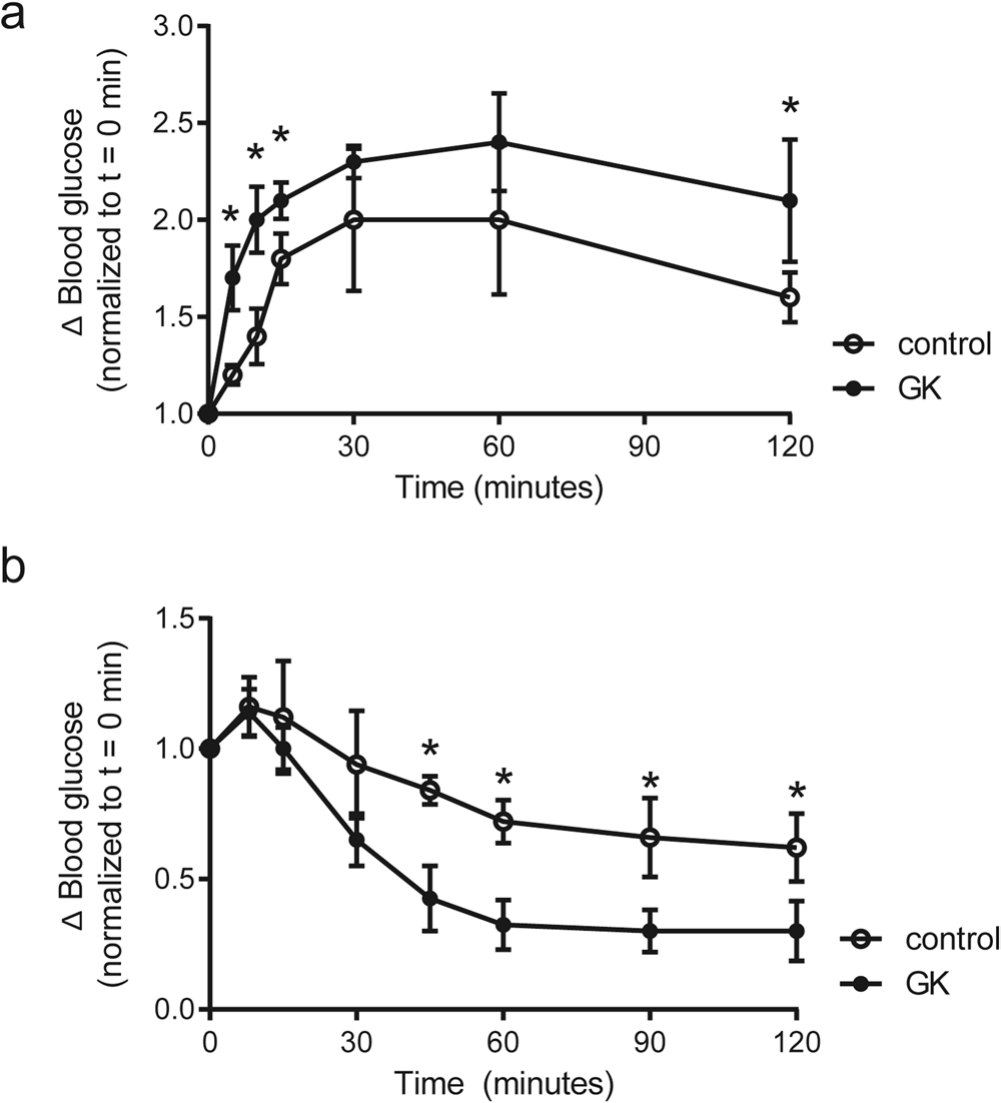
Diabetic characteristics of GK rats. The change in blood glucose levels within 2 hour duration upon (**a**) intraperitoneal injection of oral glucose (IpGTT) and upon (**b**) intraperitoneal injection of insulin (IpITT). Data are means ± SD (controls n = 5, GK n = 5). *P < 0.05 vs. controls.

To confirm the diabetic status of the animals, we determined blood parameters, and performed glucose tolerance test and insulin tolerance test. The GK rats indeed showed higher blood glucose and TG levels than controls (P < 0.0001 and P = 0.049, respectively), while insulin levels were lower in the GK rats compared with control rats (P = 0.001; Table 1). β-OHB levels were however not different between GK rats and controls (Table 1). The GK rats had impaired glucose tolerance (Fig. 1a), while administration of insulin lowered blood glucose during insulin tolerance test (Fig. 1b). The lower serum insulin levels and the characteristics shown during insulin tolerance test suggest impaired insulin secretion, consistent with the aetiology of type 2 diabetes mellitus reported in GK rats^21^. Confirming GK rats as a non-obese diabetic model, body weight was indeed lower in the GK rats than in control rats (Table 1).

**Table 1:** Body weight and blood parameters.

	Control	GK	P value
Body weight (g)	479.8 ± 30.0	403.3 ± 16.5***	<0.0001
***Fasting values***			
Blood glucose (mM)	5.3 ± 0.5	9.3 ± 1.1***	<0.0001
Blood β-OHB (mM)	1.1 ± 0.2	1.1 ± 0.3	0.580
Blood TG (mM)	2.0 ± 0.3	2.5 ± 0.7*	0.049
***Fed values***			
Blood glucose (mM)	7.6 ± 1.2	12.6 ± 3.0***	<0.0001
Blood β-OHB (mM)	0.3 ± 0.1	0.4 ± 0.1	0.540
Serum acetoacetate (mM)	0.52 ± 0.02	0.55 ± 0.02**	0.002
Total ketone bodies (mM)	0.86 ± 0.11	0.92 ± 0.08	0.185
Serum insulin (fed, μg/L)	9.3 ± 3.7	3.2 ± 1.6**	0.001
Serum glucagon (fed, pM)	54.9 ± 29.8	57.1 ± 25.5	0.867

β-OHB: β-hydroxybutyrate; TG: triglycerides; total ketone bodies: β-OHB + acetoacetate. Glucose, fasting β-OHB, and TG were measured at 20 weeks of age, while fed β-OHB, acetoacetate, insulin, and glucagon were measured at sacrifice. Data are means ± SD (control n = 10 and GK rats n = 9). *P < 0.05, **P < 0.01, ***P < 0.001.

### Hyperpolarization and lifetime of the synthesized [3-^13^C]acetoacetate allow probing ketone body utilization *in vivo*^13^C MRS

**Figure 2:**
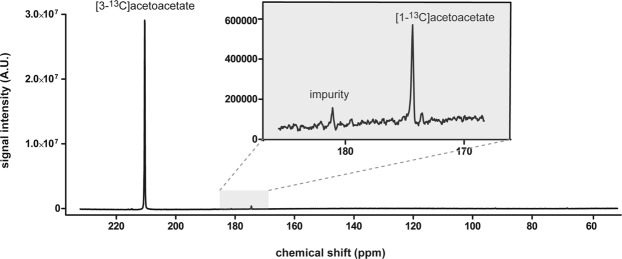
^13^C MR spectrum of 4 mM hyperpolarized [3-^13^C]acetoacetate. Upon injection of 80 mM hyperpolarized [3-^13^C]acetoacetate *in vivo*, the expected [3-^13^C]acetoacetate concentration in the blood is ~4 mM. The [3-^13^C]acetoacetate is seen at 209.9 ppm. The natural abundant [1-^13^C]acetoacetate appears at 174.6 ppm, and an impurity peak is visible at 181.0 ppm.

We first determined whether the synthesized lithium [3-^13^C]acetoacetate can be hyperpolarized with sufficient polarization and lifetime for *in vivo* application. After 120 minutes of microwave irradiation at 1.2 K, the achieved polarization was 10.3 ± 1.2% (n = 8) and T_1_ was 28.2 ± 3.1 s (n = 3). A representative hyperpolarized ^13^C MR spectrum of 4 mM lithium [3-^13^C]acetoacetate solution is shown in Fig. 2. Besides the main substrate peak at 209.9 ppm and the natural abundant [1-^13^C]acetoacetate at 174.6 ppm, an impurity peak was detected at 181.0 ppm. The impurity peak was the result of lithium [3-^13^C]acetoacetate synthesis, as this was not seen in the ^13^C NMR spectrum of the [3-^13^C]ethyl acetoacetate as the starting material (Supplementary Fig. S1). The amplitude of the [1-^13^C]acetoacetate was 1.33 ± 0.1%, and the impurity peak was 0.19 ± 0.01% of the [3-^13^C]acetoacetate signal. The impurity peak overlaps with [5-^13^C]glutamate peak *in vivo* at 181.2 ppm. However, considering that [5-^13^C]glutamate presents at a concentration range of 2 – 4% of the [3-^13^C]acetoacetate signal *in vivo*, the contribution of the impurity peak is negligible.

Acetoacetate is a major entry ketone body for energy production in the heart. The catabolism of acetoacetate generates two metabolic intermediates: (1) β-OHB, catalyzed by mitochondrial β-hydroxybutyrate dehydrogenase-1 (BDH1), and (2) acetoacetyl-CoA (AcAc-CoA), catalyzed by the rate-limiting SCOT. AcAc-CoA is then rapidly converted into acetyl-CoA by acetoacetyl-CoA thiolase (ACAT). This fuel unit is then either stored as acetylcarnitine upon catalysis by carnitine acetyltransferase (CAT), or incorporated into the tricarboxylic acid (TCA) cycle via citrate synthase (CS) mediation.

**Figure 3:**
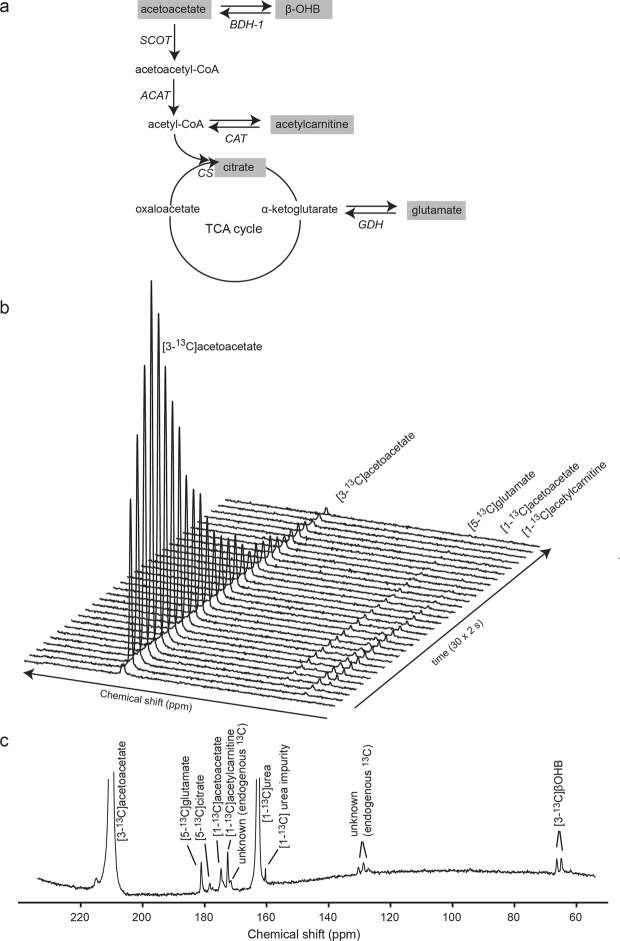
Detection of ketone metabolic products in the heart using hyperpolarized ^13^C MRS. (**a**) Metabolic fate of hyperpolarized [3-^13^C]acetoacetate. Metabolites in grey can be detected by *in vivo* ^13^C MRS in the heart. (**b**) Dynamic *in vivo* cardiac ^13^C MR spectra over 1 minute period after [3-^13^C]acetoacetate injection, with a time resolution of ~2 s. The spectra are truncated at 170 ppm. (**c**) A representative cardiac ^13^C MR spectrum, summed over 60 seconds (30 spectra) upon [3-^13^C]acetoacetate arrival, with [1-^13^C] urea used as an external reference. β-OHB: β-hydroxybutyrate, BDH1: 3-hydroxybutyrate dehydrogenase-1, SCOT: succinyl-CoA:3-ketoacid-coenzyme A transferase, ACAT: acetoacetyl-CoA thiolase, CAT: carnitine-acylcarnitine translocase, CS: citrate synthase, GDH: glutamate dehydrogenase, TCA:  tricarboxylic acid.

For *in vivo* application, we administered hyperpolarized [3-^13^C]acetoacetate intravenously then followed the evolution of the ^13^C MR spectra within ~2 minute period, which revealed the incorporation of the ^13^C label from [3-^13^C]acetoacetate into its metabolic products (Fig. 3a,b). The following resonances were detected: [3-^13^C]acetoacetate peak at 209.9 ppm, the natural abundant [1-^13^C]acetoacetate at 174.6 ppm, and the downstream metabolites: [1-^13^C]acetylcarnitine at 172.6 ppm, [5-^13^C]glutamate at 181.2 ppm, and [5-^13^C]citrate at 178.4 ppm. A representative ^13^C MR spectrum of the summed spectra over 60 seconds upon acetoacetate arrival is shown in Fig. 3c. In addition to the above-mentioned resonances, [3-^13^C]β-OHB appears as a doublet at 68.5 ppm. Resonances at 127 ppm, 128.6 ppm, 130.2 ppm, and 171.5 ppm also appear in the baseline ^13^C MR spectra prior to injection of [3-^13^C]acetoacetate (Supplementary Fig. S2), which indicates that these resonances originate from endogenous ^13^C in the tissue, potentially lipids^22,23^.

### Increased ketone body utilization in diabetic rats as observed by ^13^C MRS upon hyperpolarized [3-^13^C]acetoacetate administration

**Figure 4:**
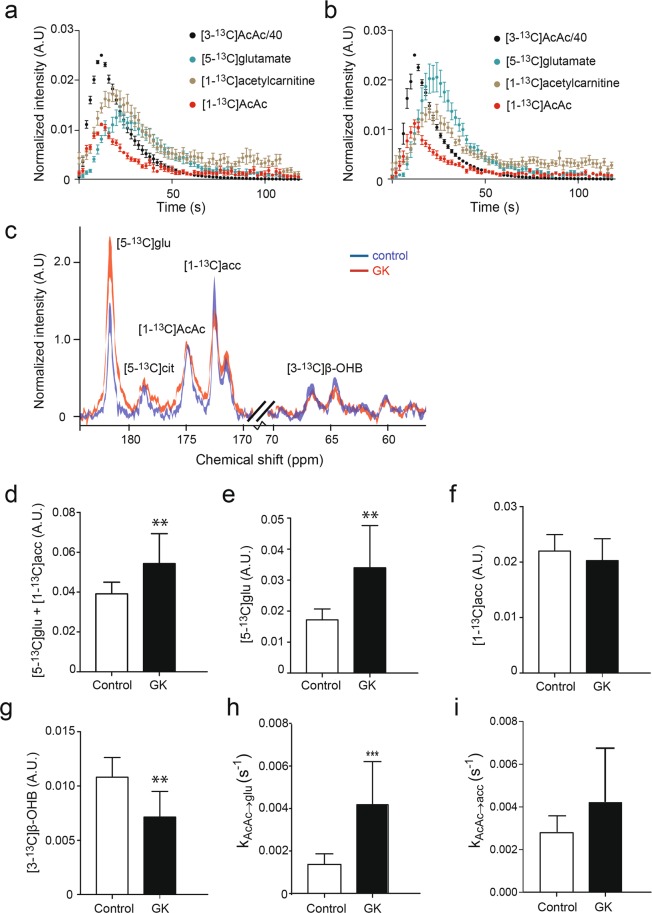
Myocardial ketone body utilization in GK rats. The detection of [3-^13^C]acetoacetate, [1-^13^C]acetoacetate, [5-^13^C]glutamate, and [1-^13^C]acetylcarnitine over 2 minutes upon [3-^13^C]acetoacetate injection in (**a**) controls and (**b**) GK rats. (**c**) Representative cardiac ^13^C MR spectra from a control and a GK rat. The quantification of (**d**) [5-^13^C]glutamate + [1-^13^C]acetylcarnitine, (**e**) [5-^13^C]glutamate, (**f**) [1-^13^C]acetylcarnitine, and (**g**) [3-^13^C]β-OHB. Metabolic conversion rates for (**h**) [3-^13^C]acetoacetate to [5-^13^C]glutamate exchange and (**i**) [3-^13^C]acetoacetate to [1-^13^C]acetylcarnitine exchange. Data are means ± SD (except for (**a**) and (**b**): mean ± SEM), and normalized to [3-^13^C]acetoacetate (controls n = 10, GK n = 9; except for (i) GK n = 8). AcAc: acetoacetate, acc: acetylcarnitine, cit: citrate, glu: glutamate. *P < 0.05, **P < 0.01, ***P < 0.001 vs. controls.

Figure 4a,b show the time course of [3-^13^C]acetoacetate, [5-^13^C]glutamate, and [1-^13^C]acetylcarnitine, and [1-^13^C]acetoacetate for 2 minutes immediately upon injection in control and diabetic GK rats, respectively. Figure 4c shows the comparison of ^13^C MR spectra between a diabetic GK rat and a control rat. We observed that the total products of ketone body utilization (i.e. [5-^13^C]glutamate + [1-^13^C]acetylcarnitine) were higher in the diabetic rats compared with controls (P = 0.009; Fig. 4d). Downstream in the ketone oxidation pathway, the production of [5-^13^C]glutamate was higher in the diabetic rats than in controls (P = 0.002; Fig. 4e). Glutamate presents in high equilibrium with a TCA cycle product, α-ketoglutarate, and as such, it is often used to indicate ^13^C carbon flow into the TCA cycle^24^. The [5-^13^C]citrate can also be used to assess the incorporation of ^13^C carbon into the TCA cycle; however, the [5-^13^C]citrate signal was too low for accurate spectral fitting. The production of [1-^13^C]acetylcarnitine was similar between groups (P = 0.30; Fig. 4f). Taken together, these results indicate an increased myocardial ketone body utilization in the diabetic rats, which was directed more towards oxidation in the TCA cycle, rather than incorporation into acetylcarnitine. Furthermore, we were also able to detect the production of [3-^13^C]β-OHB, which was lower in the diabetic rats than in control rats (P = 0.001; Fig. 4g), suggesting lower BDH1 activity and mitochondrial redox state in the diabetic GK rats.

In addition, we also performed kinetic modelling to calculate metabolic conversion rates from [3-^13^C]acetoacetate to the metabolic products. In agreement with data of metabolic ratios, metabolic conversion rates for [3-^13^C]acetoacetate to [5-^13^C]glutamate exchange were higher in GK rats than in controls (P = 0.0004; Fig. 4h), while those for [3-^13^C]acetoacetate to [1-^13^C]acetylcarnitine exchange were similar between groups (P = 0.11; Fig. 4i). The metabolic conversion rates obtained by kinetic modelling correlated well with the data of metabolic ratios (for [5-^13^C]glutamate: r = 0.79, P < 0.0001; for [1-^13^C]acetylcarnitine r = 0.51, P = 0.33, Supplementary Fig. S3).

### Increased ketone body utilization in diabetic rats correlated with SCOT activity

**Figure 5:**
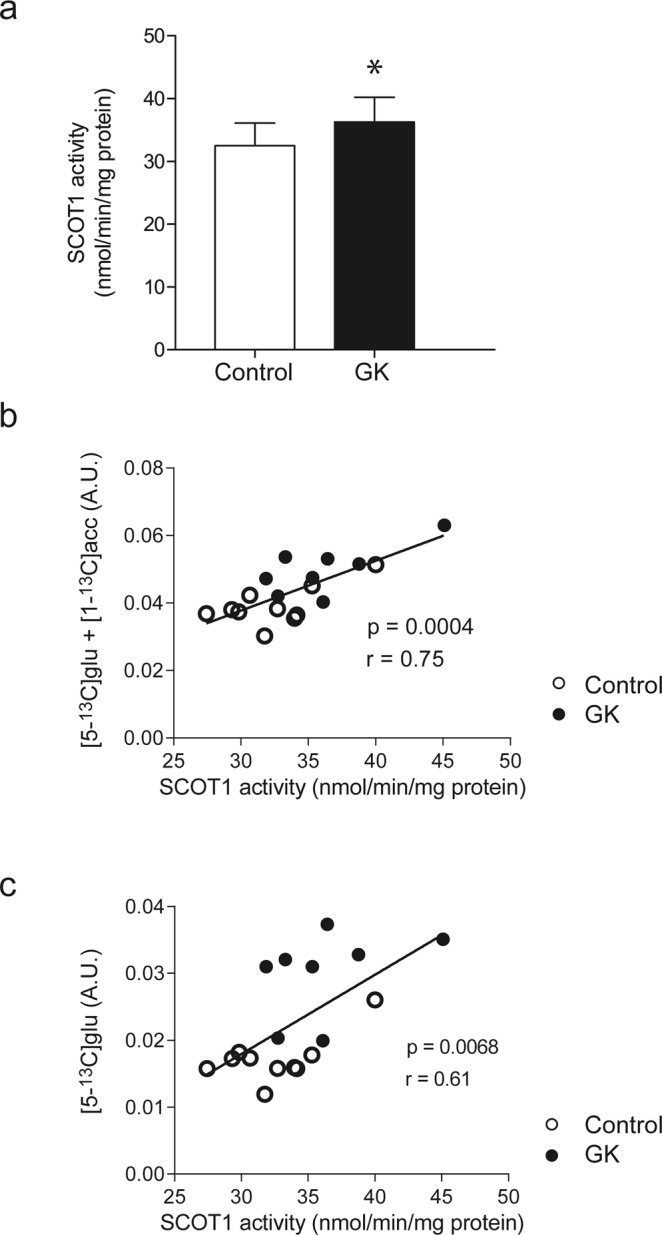
Increased ketone body utilization in GK rats was associated with increased SCOT activity. (**a**) SCOT activity. Correlation between (**b**) SCOT activity and ([5-^13^C]glutamate + [1-^13^C]acetylcarnitine), and between (**c**) SCOT activity and [5-^13^C]glutamate. Data are means ± SD (controls n = 10, GK n = 9, except for the correlation n = 8). One outlier was not included in the correlation, see Supplementary Fig. S4 for complete data. Acc: acetylcarnitine, glu: glutamate. *P < 0.05 vs. controls.

We then tested whether the increased ketone body utilization in the diabetic heart was associated with increased myocardial SCOT activity. Indeed, SCOT activity was higher in the diabetic rats compared with control (P = 0.045; Fig. 5a), which is in agreement with the *in vivo* ^13^C MRS results. Furthermore, SCOT activity correlated significantly with the total of ketone body utilization products (i.e. [1-^13^C]acetylcarnitine + [5-^13^C]glutamate) (r = 0.75, P = 0.0004; Fig. 5b) as well as with the product of ketone oxidation (i.e. [5-^13^C]glutamate) (r = 0.61; P = 0.0068; Fig. 5c).

### Cardiac hypertrophy and reduced ejection fraction in the diabetic rats correlated with increased ketone body utilization

**Figure 6:**
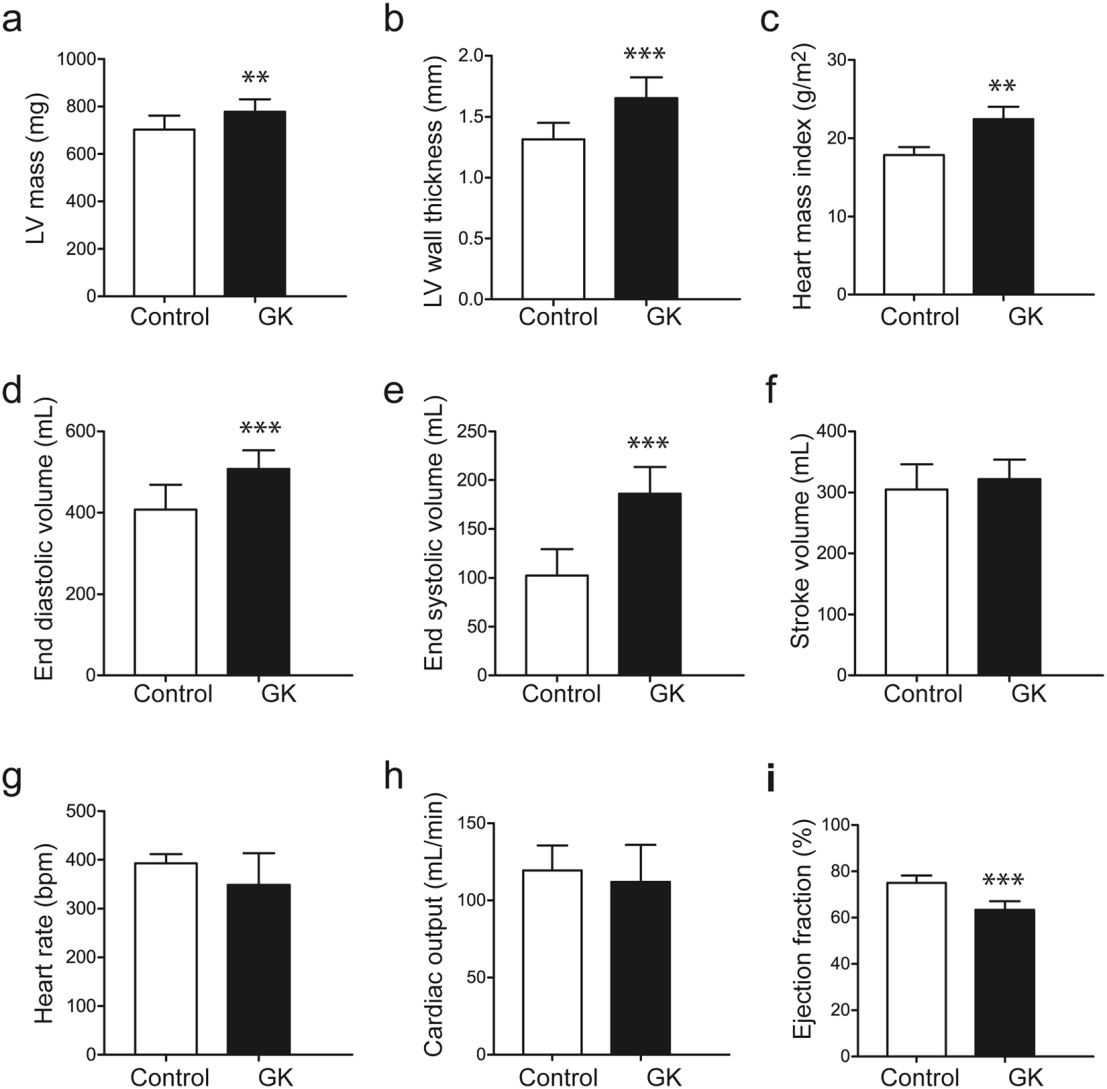
GK rats exhibited cardiac hypertrophy and dysfunction. (**a**) LV mass, (**b**) diastolic wall thickness, (**c**) heart mass index (post-mortem), (**d**) end diastolic volume, (**e**) end systolic volume, (**f**) stroke volume, (**g**) heart rate, (**h**) cardiac output, and (**i**) ejection fraction. Data are means ± SD (controls n = 10, GK n = 9). **P < 0.01, ***P < 0.001 vs. controls.

**Figure 7:**
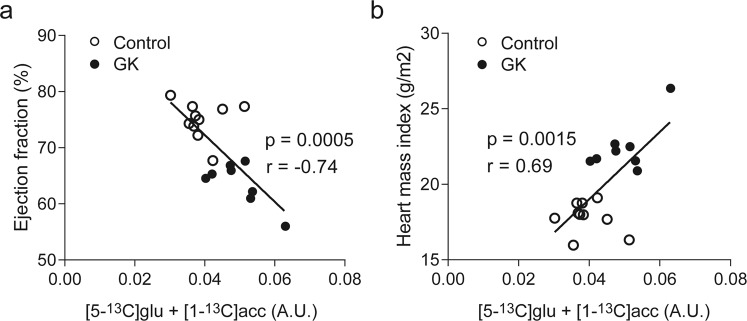
Total of ketone body utilization products correlated with cardiac hypertrophy and function. Correlation between (**a**) ([5-^13^C]glutamate + [1-^13^C]acetylcarnitine) and cardiac function, and (**b**) between ([5-^13^C]glutamate + [1-^13^C]acetylcarnitine) and heart mass index. Controls n = 10, GK n = 8 (one outlier was not included, see Supplementary Fig. S4 for complete data). Acc: acetylcarnitine, glu: glutamate.

To investigate whether the modulation in cardiac metabolism in the diabetic rats was accompanied by an alteration in cardiac function, we performed cinematic MRI to assess cardiac function. At 24 weeks of age, the diabetic rats exhibited increased LV mass and LV wall thickness (P = 0.008 and P = 0.0002 vs. control, respectively; Fig. 6a,b), which indicate cardiac hypertrophy. This is in agreement with the higher post-mortem heart mass index in the diabetic rats (P < 0.0001 vs. control; Fig. 6c). The hypertrophy in the diabetic rats was also evidenced by higher end diastolic volume and end systolic volume (P = 0.0009 and P = < 0.0001 vs. control, respectively; Fig. 6d,e), while stroke volume was maintained at similar levels as controls (P = 0.34; Fig. 6f). Heart rate and cardiac output were also not significantly different between diabetic rats and control rats (P = 0.054 and P = 0.43, respectively; Fig. 6g,h). Notably, ejection fraction was lower in diabetic rats than in control rats (P < 0.0001; Fig. 6i). Interestingly, we observed that the total of ketone body utilization products strongly correlated with ejection fraction (r = −0.74, P = 0.0005; Fig. 7a) and with heart mass index (r = 0.69, P = 0.0015; Fig. 7b).

## Discussion

Myocardial ketone body metabolism has recently gained attention as studies have shown its potential relevance in nutrient-deprived^8^, diabetes^25^, or pathological states such as heart failure^3,4^. However, methods to detect and measure *in vivo* ketone body utilization non-invasively in real-time with high specificity are limited. In this work, we synthesized [3-^13^C]acetoacetate as a metabolic probe to study ketone metabolism *in vivo* using hyperpolarized ^13^C MRS. The detection of downstream metabolites [5-^13^C]glutamate, [1-^13^C]acetylcarnitine, and [5-^13^C]citrate in ^13^C MR spectra within a minute window upon [3-^13^C]acetoacetate injection provides evidence that the hyperpolarized [3-^13^C]acetoacetate is taken up into the myocardium and catabolized into metabolic intermediates involved in the ATP production. *In vivo*, we demonstrated higher ^13^C incorporation into [5-^13^C]glutamate in the diabetic rats compared with control rats, indicating higher myocardial ketone body oxidation in the diabetic rats. This result was validated by higher activity of SCOT, the rate-limiting enzyme in ketolysis, in the myocardium tissue of diabetic rats.

Diabetes has been associated with alterations in ketone body metabolism. Increased levels of ketone body production in GK rats as measured in urine samples has been reported in a previous study^26^, as well as altered expression of genes involved in myocardial ketone metabolism^27,28^. To our knowledge, this study is the first to report that myocardial ketone oxidation was increased in non-obese diabetic Goto-Kakizaki rats, despite similar circulating ketone body levels as compared to controls. It is noteworthy that alterations in myocardial ketone utilization may occur without alterations in ketone body availability in the blood, as general assumption in myocardial ketone utilization is often based on circulating ketone levels. We further show that the increased myocardial ketone body oxidation in the diabetic rats was correlated with cardiac hypertrophy (i.e. increased cardiac index) and lower ejection fraction, indicating the development of diabetic cardiomyopathy in the diabetic GK rats. While large body of literature focused on the role of myocardial fatty acids or glucose oxidation in diabetic cardiomyopathy^1,29,30^, our results provide evidence that an alteration in myocardial ketone body utilization may also be a contributing factor.

In agreement with our finding, myocardial ketone body oxidation has been shown to be increased in advanced heart failure^3,4^. However, it is currently not clear whether the increase in ketone body oxidation in failing heart is an adaptive or maladaptive mechanism. A previous study showed that mice with cardiac-specific SCOT-deficiency had an exacerbated increase in LV mass and reduction in ejection fraction after 8 weeks of pressure-overload^9^, suggesting that increased ketone oxidation may be important in heart failure settings. In agreement, a study in ischemia-reperfusion in rats showed that administration of β-OHB in fasted animals reduced myocardial infarct size and apoptosis^6^, although myocardial ketone oxidation was not measured. More excitingly, a recent clinical trial with empagliflozin, a sodium/glucose co-transporter-2 (SGLT-2) inhibitor, showed an improved cardiovascular outcome in diabetic patients with empagliflozin treatment^31^. An increase in plasma ketone body concentration was observed after empagliflozin treatment, leading to a hypothesis that the cardio-protective effects of empagliflozin was attributed to increased cardiac efficiency, which might be associated with increased utilization of ketone bodies as ‘super fuel’^32,33^. However, this ‘fuel hypothesis’ has recently been challenged, proposing that sustained ketone body oxidation may actually be detrimental^34^. This debate remains open as data on myocardial ketone body oxidation is currently lacking, due to lack of available techniques to probe ketone body oxidation *in vivo*. Here, our novel method to assess myocardial ketone body utilization *in vivo* will be proven beneficial in the endeavour to understand ketone body metabolism.

Hyperpolarized ^13^C MRS using ^13^C acetoacetate could also provide insight into mitochondrial redox state^35^. Acetoacetate and β-OHB are a redox pair, and the formation of β-OHB from acetoacetate is regulated by and proportional to the NADH/NAD^+^ ratio^35,36^. Therefore, the lower ratio of [3-^13^C]β-OHB/[3-^13^C]acetoacetate in the diabetic GK rats suggests lower mitochondrial NADH/NAD^+^ and redox state, which is in agreement with mitochondrial redox impairment generally found in diabetic heart^37^. A recent study using hyperpolarized [1,3-^13^C_2_]acetoacetate showed an increase in mitochondrial redox state following metformin treatment, as indicated by increased ratio of [1-^13^C]β-OHB/[1,3-^13^C_2_]acetoacetate in rat kidney *in vivo*^20^.

Recently, another ^13^C acetoacetate probe to study ketone body metabolism using hyperpolarized ^13^C MRS has been reported, by labelling the first carbon position rather than the third carbon position^19^. The reported T_1_ value of [1-^13^C]acetoacetate at 11.7 T is similar to the T_1_ value of the [3-^13^C]acetoacetate at 9.4 T in the present study. Compared to [1-^13^C]acetoacetate, [3-^13^C]acetoacetate has an advantage that its chemical shift (209.9 ppm) is away from the chemical shift of the metabolic products, which would make spectral fitting and quantification more accurate. Another hyperpolarized ^13^C-acetoacetate probe that has been reported was [1,3-^13^C_2_]acetoacetate, for applications in kidney^20^. A doubly labelled ketone body probe may be preferred because of the increase in sensitivity compared to a single labelled probe, considering that ketone body metabolic products are typically present in a low concentration.

There are several considerations for data interpretation using this method. First, as the ^13^C MR spectra were acquired using surface coil localization, the hyperpolarized [3-^13^C]acetoacetate and [1-^13^C]acetoacetate signal were probably derived from both the myocardium and the blood pool, while [5-^13^C]glutamate, [5-^13^C]citrate, and [1-^13^C]acetylcarnitine signals originated mainly from myocardial metabolism. Therefore, the normalization to the substrate signal (i.e. [3-^13^C]acetoacetate) likely underestimated the levels of myocardial ketone oxidation. Also, one may need to consider different contributions of blood signal for example when comparing groups with different cardiac output. In the present study, cardiac output was not different between the diabetic rats and control rats.

Another issue with surface coil localization is the possibility of signal contamination from other organs, in our case, liver (Supplementary Fig. S5). We performed separate hyperpolarized ^13^C MRS experiments using [3-^13^C]acetoacetate, in which the liver was positioned on top of the coil. We observed that the production of [5-^13^C]glutamate was 0.39 ± 0.13% (n = 3) of the [3-^13^C]acetoacetate signal (Supplementary Fig. S6), which was much lower compared to typical values obtained from cardiac ^13^C MRS experiments (~2–4%, Fig. 4e). Considering that during cardiac ^13^C MRS experiments, the liver was not positioned in the most sensitive area of the coil and only part of the liver was covered by the coil, we believe that the contribution of the liver signal was minimal. To circumvent the issues associated with surface coil localization, a high resolution ^13^C spectroscopic imaging may be performed to spatially resolve the signal; however, a relatively short T_1_ value and low signal-to-noise ratio (SNR) of the metabolite signals associated with ketone body metabolism would have to be taken into consideration. Higher polarization at 5 T is an improvement that we are working on to increase the SNR.

Another consideration is that there may be an uptake competition between the injected hyperpolarized ^13^C labelled acetoacetate and the endogenous (unlabelled) acetoacetate, which may result in underestimation of ketone oxidation rate. However, since the concentration of injected acetoacetate was much higher than the endogenous acetoacetate in the blood, such uptake competition is expected to be minimal. For example, in the present study, compared with the endogenous acetoacetate concentration, the calculated acetoacetate concentration in the blood after injection was ~7 times higher (Supplementary Table S1).

‘Pseudoketogenesis’ may influence the quantification of ketone oxidation rate *in vivo*^38,39^. In extrahepatic tissues, there may be production of endogenous acetoacetate from acetoacetyl-CoA (derived from fatty acids) or acetyl-CoA (derived from glucose), via reversal of SCOT or ACAT activity^38,39^. This production of unlabelled endogenous acetoacetate would then dilute the concentration of ^13^C labelled acetoacetate, which could complicate the quantification of ketone oxidation rate. The concept of pseudoketogenesis has been demonstrated *in vivo* using bolus injection of [3,4-^13^C_2_]acetoacetate in hepatectomized dogs^39^. During 60 minutes after [3,4-^13^C_2_]acetoacetate injection, molar percent of ^13^C enrichment (MPE; i.e. ^13^C labelled acetoacetate concentration) in the blood indeed decreased over time^39^. As hepatic ketogenesis was expected to be absent in the hepatectomized dogs, any dilution in the ^13^C labelled acetoacetate concentration after the injection of [3,4-^13^C_2_]acetoacetate could be attributed to the pseudoketogenesis, rather than the hepatic ketogenesis. Indeed, the decrease in MPE over time became even more prominent when glucose oxidation was stimulated using dichloroacetate^39^. However, during the first 2 minutes after the bolus injection, the dilution in MPE was less than ~5% (in both cases with or without dichloroacetate)^39^, suggesting that the effects of pseudoketogenesis in the time frame relevant for hyperpolarized ^13^C MRS experiments may be minimal.

Pseudoketogenesis has also been shown to lead to ^13^C (or ^14^C) enrichment in carbon positions other than initially labelled^38,39^. Therefore, [1-^13^C]acetoacetate peak observed in the ^13^C MR spectrum after injection of [3-^13^C]acetoacetate could have also been contributed by label exchange via SCOT and ACAT activity, in addition to being a natural abundance present in the lithium [3-^13^C]acetoacetate dissolution. The amplitudes of [1-^13^C]acetoacetate peak in the present study (controls: 1.3 ± 0.1%, GK: 1.1 ± 0.2%) were however very close to the [1-^13^C]acetoacetate amplitude in the lithium [3-^13^C]acetoacetate dissolution (1.33 ± 0.1%), which suggests that the contribution of pseudoketogenesis to the [1-^13^C]acetoacetate may be negligible.

Another point of consideration is the injected dose of acetoacetate. The limit on the amount of [3-^13^C]acetoacetate that can be injected may be related to potential adverse side effects of injecting substrates at a high concentration. We did not observe adverse physiological effects using the dose in the present study (0.24 mmol/kg, resulting in ~4 mM acetoacetate in the blood). Other studies with ^13^C acetoacetate in rats reported an injected dose similar to ours (2 mL, 40 mmol/L ≈ 0.26 mmol/kg assuming rat weight of 300 g)^19^ or lower (2.2 mL, 20 mmol/L ≈ 0.15 mmol/kg assuming rat weight of 300 g)^20^. However, a study in dogs reported a higher  injected dose of 0.8 mmol/kg^39^. It has been reported that at a concentration higher than 2 mM, acetoacetate infusion exerts a transient stimulatory effect on insulin secretion in man, which abates after 10-15 minutes^40^. Insulin secretion may affect SCOT activity. However, the ^13^C MRS acquisition is only two minutes and it begins immediately upon injection of hyperpolarized acetoacetate. While the amount of insulin secreted as a result of this infusion is unknown, its effect on SCOT activity is expected to be negligible given the relatively short acquisition window. Nonetheless, it is part of future work to characterize this phenomenon.

Lastly, there may be a concern of lithium toxicity because the administration of lithium [3-^13^C]acetoacetate resulted in a blood lithium concentration of ~3.75 mM. However, serum lithium concentration at 90 minutes after the injection was reduced to 0.24–0.35 mM (Supplementary Table S2), which is within a safe therapeutic range of 0.3–1.3 mM^41^.

In conclusion, we demonstrated the potential of hyperpolarized [3-^13^C]acetoacetate in probing myocardial ketone body oxidation *in vivo*. In the diabetic GK rats, we observed that myocardial ketone utilization and oxidation were higher compared with controls, which was validated with higher enzymatic activity of the rate-limiting SCOT protein. The higher myocardial ketone utilization and oxidation in diabetic rats were correlated with cardiac hypertrophy and lower ejection fraction, suggesting a tight coupling between ketone body metabolism and cardiac health. The hyperpolarized MRS with [3-^13^C]acetoacetate is a non-invasive technique, which eliminates the need for biopsy or termination of animals for tissue collection at desired time point. As such, it allows longitudinal assessment of myocardial ketone body oxidation in future research in the diseased heart.

## Materials and Methods

The datasets generated during and/or analysed during the current study are available from the corresponding author on reasonable request.

### Animals

Male non-obese diabetic Goto-Kakizaki (GK/MolTac; n = 9) rats bred by Taconic (New York, US) were ordered from *InVivos* (Singapore). Male wild-type Wistar Han rats (n = 10) were purchased from *InVivos* (Singapore) and served as controls. The animals were housed under controlled temperature (25 °C) and humidity (60%) with a 12:12-h dark-light cycle, with *ad libitum* access to food and water. At 20 weeks of age, blood parameters (glucose, β-OHB, and triglycerides) were determined under fed and fasted condition. To test peripheral insulin and glucose tolerance, intraperitoneal insulin tolerance test (IpITT) and glucose tolerance test (IpGTT) were performed (n = 5 each group). At 24 weeks of age, the animals underwent ^13^C MRS and MRI to determine myocardial ketone body utilization and cardiac function, under fed condition. The animals were anaesthetized with isoflurane (3% for induction; 1.5–2% for maintenance) in medical air and oxygen at a flow rate of 2.0 L/min and 0.5 L/min, respectively. A catheter was inserted into the tail vein for intravenous administration of the hyperpolarized [3-^13^C] acetoacetate inside the MRI scanner. At 25 weeks of age, the animals were sacrificed. Blood and organs were collected and stored at −80 °C for biochemical analysis. The overview of experiments is provided in Supplementary Fig. S7. All experimental protocols involving animals were approved by A*STAR Institutional Animal Care and Use Committee (IACUC, #151078), and carried out in accordance with the guidelines and regulations of A*STAR IACUC.

### Lithium [3-^13^C]acetoacetate synthesis, polarization and dissolution

The lithium [3-^13^C]acetoacetate probe has been filed for patent protection by A*STAR (patent application number: 10201609057Q).

#### Chemical synthesis

The chemical synthesis of lithium [3-^13^C]acetoacetate involves the saponification of [3-^13^C]ethyl acetoacetate which was performed according to Hall^42^. The starting materials were 1 gram of [3-^13^C]ethyl acetoacetate (Cambridge Isotope Laboratories, US), 4.8 mL of water, and 1.9 mL of 4 mol/L lithium hydroxide (Sigma Aldrich, US). The yield obtained was 40%. The purity of [3-^13^C]acetoacetate was assessed by ^13^C-NMR and hyperpolarized ^13^C-MRS (below).

#### Polarization and dissolution

Approximately 50 mg of lithium [3-^13^C]acetoacetate, doped with 5.1 mg of trityl-radical (OXO63, GE Healthcare, US), 246 μl of gadoterate meglumine (1.2 mmol/L, Dotarem®, Guerbet, France) and 93 μL of DMSO (25% v/v) was prepared. Total volume is approximately 370 μL, resulting in 1.23 mol/L of [3-^13^C]acetoacetate, 9.0 mmol/L of OXO63, and 0.80 mmol/L of gadolinium. Then, 200 μL of the sample was hyperpolarized in a polarizer (Hypersense, Oxford Instruments, UK), with 120 min of microwave irradiation at 94.097 GHz. The polarized sample was subsequently dissolved in 3 mL of pressurized and heated Tris-EDTA buffer with pH 7.80, to yield a solution of 80 mmol/L hyperpolarized [3-^13^C]acetoacetate with a polarization of 10% and physiological temperature and pH.

### Determination of polarization and T_1_

Liquid state polarization was determined using MQC NMR analyzer (Oxford Instruments, UK). T_1_ was determined in hyperpolarized ^13^C MRS experiments at 9.4 T MR scanner (Bruker, Germany). A 20 mL syringe was placed on top of a dual-tuned (^1^H/^13^C) surface coil (^13^C diameter: 40 mm, ^1^H diameter: 50 mm; Rapid Biomedical, Germany) and first filled with dissolution buffer for shimming. Subsequently, 0.5 mL of the buffer was replaced by dissoluted lithium [3-^13^C]acetoacetate, resulting in a concentration of 4 mmol/L in the syringe. A series of ^13^C MR pulse-acquire spectroscopy sequence was then performed (TR, 2 s; sweep width, 18,116 Hz (180.08 ppm); acquired points, 4,096; frequency centered at 190 ppm from tetramethylsilane (TMS) standard), using excitation flip angles (FA) of 5°, 10°, 15°, and 20°, with 5 repetitions for each FA, and repetition time (TR) of 1 s. Peak amplitudes were fitted using AMARES^43^ in the jMRUI software package (http://www.jmrui.eu)^44,45^. The exponential decay constants (K) of the [3-^13^C]acetoacetate amplitudes were determined for each excitation FA. T_1_ was then calculated from data fitting of the following equation^46^: $K(FA)=\frac{-\mathrm{log}(\cos (c\ast FA))}{TR}+\frac{1}{{T}_{1}}$. The derivation of this equation is provided in Supplementary Materials.

### Ketone body utilization in the heart *in vivo* measured by hyperpolarized ^13^C-MRS

^13^C MRS experiments were performed using a 9.4 T horizontal bore MR scanner interfaced to a Avance III HD console (Biospec, Bruker, Germany), under fed condition. Rats were in a prone position, with the heart located directly above a dual-tuned (^1^H/^13^C) rat surface coil (^13^C diameter: 40 mm, ^1^H diameter: 50 mm; Rapid Biomedical, Germany). ^13^C urea phantom was positioned next to the animal (Supplementary Fig. S5) for ^13^C power calibration. Anatomical images were acquired using ^1^H FLASH (TE/TR, 8.0/100.0 ms; matrix size, 128 × 128; FOV, 40 × 40 mm; slice thickness, 1.5 mm; excitation flip angle, 30°). A cardiac-triggered and respiratory-gated shim was performed to result in the proton linewidth of approximately 160 Hz. A cardiac-triggered ^13^C MR pulse-acquire spectroscopy sequence was initiated immediately before injection. Then, hyperpolarized [3-^13^C] acetoacetate (0.75–1.05 mL; 0.240 mmol/kg body weight) was injected via tail vein at a rate of 6 mL/min. Sixty individual heart spectra were acquired over 2 minutes after injection (TR, 2 s; excitation flip angle, 25°; sweep width, 18,116 Hz (180.08 ppm); acquired points, 4,096; frequency centered at 190 ppm from tetramethylsilane (TMS) standard).

In a separate set of wild-type animals (n = 3), ^13^C MRS experiments were performed by positioning the liver on top of the coil, to assess possible contamination from the liver during cardiac ^13^C MRS. The parameters were similar as above, except sweep width: 8,012 Hz (80 ppm).

### ^13^C-MRS data analysis

Cardiac ^13^C MR spectra were analyzed using AMARES^43^ in the jMRUI software package (http://www.jmrui.eu)^44,45^. Spectra were baseline and DC offset-corrected based on the last half of acquired points. To quantify myocardial ketone metabolism, spectra were summed over the first 30 spectra (i.e. 60 seconds) upon acetoacetate arrival. Peaks corresponding to [3-^13^C]acetoacetate and its metabolic derivatives (i.e., [5-^13^C]glutamate, [1-^13^C]acetoacetate, and [1-^13^C]acetylcarnitine) were fitted with prior knowledge assuming a Lorentzian line shape, peak frequencies, relative phases, and linewidths. The fitted amplitudes were then normalized to the amplitude of [3-^13^C]acetoacetate. To calculate metabolic conversion rates from [3-^13^C]acetoacetate to the metabolic derivatives, dynamic metabolic data were fitted using kinetic modelling, adapted from the work by Atherton *et al*.^47^ (see Supplementary Materials for details).

### *In vivo* assessment of cardiac function

Cardiac MRI measurements were performed at a 9.4 T preclinical MRI system (Biospec, Bruker, Germany), in the same session as the ^13^C MRS, under fed condition. ECG-triggered respiratory-gated cine-MRI was acquired in 7–8 contiguous short-axis slices (slice thickness: 1.5 mm) covering the entire heart. The imaging parameters were FOV 40 × 40 mm^2^, matrix size 128 × 128, TE/TR 1.4/8.0 millisecond, 30° SLR excitation pulse, 18–23 frames/cardiac cycle, total experiment time ~20 minutes. Cine-MRI images were exported into DICOM format and loaded into MIPAV (NIH, US) for subsequent region-of-interest analysis. For LV volumes and mass measurements, end-diastolic and end-systolic frames were selected according to maximal and minimal ventricular volume. In both frames, the epicardial and LV cavity border were outlined manually. The difference in area between these two ROIs multiplied by the slice thickness of 1.5 mm yielded the myocardial volume. LV mass was calculated as myocardial volume multiplied by the specific gravity of 1.05 g/cm^3^ ^48^. The end-systolic (ESV) and end-diastolic (EDV) volumes were calculated as the LV cavity area multiplied by the slice thickness. Stroke volume (SV = EDV – ESV), ejection fraction (EF = SV/EDV), and cardiac output (CO = SV × heart rate) were then calculated as measures of cardiac function^49^.

### Determination of blood parameters

Blood samples (4 µL) were collected from the tail at fed condition and after 16.5 hours of fasting. Blood glucose and triglyceride levels were determined using Accu-Check Advantage glucometer and Accutrend Plus meter, respectively (Roche, Germany). Blood ketone levels were measured using Freestyle Optimum ketone meter (Abbott Laboratories, US). Serum insulin, glucagon, and acetoacetate levels were determined in blood samples collected at sacrifice. Blood samples were collected in micro-collection tubes (BD, US) with serum separator additive and left to clot for 30 min at room temperature. Thereafter, serum was collected after centrifugation at 15,000 *g* for 2 min. Serum insulin and glucagon levels were determined using ultrasensitive mouse insulin ELISA and glucagon ELISA kits, respectively (Mercodia, Sweden). Serum acetoacetate levels were determined using acetoacetate colorimetric assay kit (BioVision, Inc., US). Serum lithium levels were determined using lithium assay kit #LI01ME (Metallogenics, Japan), in serum collected at 90 minutes after [3-^13^C]acetoacetate injection.

### Intraperitoneal glucose tolerance test (IpGTT)

Glucose tolerance test was determined by measuring the blood glucose levels before and at 5, 10, 15, 30, 60, and 120 minutes after intraperitoneal injection of glucose solution (1 g/kg body weight). The animals were fasted for 16.5 hours prior to the experiments.

### Intraperitoneal insulin tolerance test (IpITT)

The response to insulin was determined by measuring the blood glucose levels before and at 8, 15, 30, 45, 60, 90, and 120 after intraperitoneal injection of insulin solution (1U/kg body weight), under fed condition.

### Determination of SCOT activity

The assay to determine SCOT activity was performed as described previously^50^. Briefly, 2 ml of reaction mix (50 mM Tris buffer (pH 8.5), 0.2 mM succinyl-CoA, 5 mM lithium acetoacetate, 5 mM MgCl_2_ and 5 mM iodoacetamide) was contained in a silica cuvette (1 cm). 400 µg protein from the tissue homogenate was added to the reaction mix. The rate of increase in absorbance at 313 nm was measured for 2 min by an absorbance reader (Infinite® M200, Tecan, Switzerland).

### Statistical analysis

All statistical analysis was performed with the Graphpad Prism (GraphPad Software, US). The data were presented as means ± SD. Statistical significance in hyperpolarized ^13^C metabolite signal ratios and *ex-vivo* cardiac enzyme activity comparisons were assessed by using a two-tailed unpaired Student’s t-test. Correlations between parameters were assessed using a Pearson’s correlation. Statistical significance in the IpGTT and IpITT curve was determined using the t-test using the Holm-Sidak method to correct for multiple comparisons. The significance was set at P < 0.05.

## Supplementary information

Supplementary Materials
