# Supplementary material for: A new hyperpolarized 13C ketone body probe reveals an increase in acetoacetate utilization in the diabetic rat heart
Source: Sci Rep. 2019 Apr 2;9:5532. doi: 10.1038/s41598-019-39378-w (PMC6445118; doi:10.1038/s41598-019-39378-w)
Supplement: Supplementary file 1 — Supplementary Materials [file 41598_2019_39378_MOESM1_ESM.docx]

**A new hyperpolarized ^13^C ketone body probe reveals an increase in acetoacetate utilization in the diabetic rat heart**

*Supplementary Materials*

Desiree Abdurrachim^1^, Chern Chiuh Woo^1^, Xing Qi Teo^1^, Wei Xin Chan^1^, George K. Radda^2^, Philip Teck Hock Lee^1^*

^1^Functional Metabolism Group, Singapore Bioimaging Consortium, Agency for Science, Technology, and Research, Singapore

^2^Department of Physiology, Anatomy and Genetics, University of Oxford, Oxford, United Kingdom

*Corresponding author:

Philip Teck Hock Lee, PhD

Singapore Bioimaging Consortium

Agency for Science, Technology, and Research

11 Biopolis Way #02-02, Singapore 138667

Tel: 65-64788722, Fax: 65-64789957

Email: Philip_Lee@sbic.a-star.edu.sg

Running title: Myocardial ketone utilization in diabetic rats

Keywords: ketone body metabolism, diabetes, ^13^C hyperpolarized MRS

**Supplementary Materials & Methods**

***Derivation of T1 determination***

Following dissolution, hyperpolarized ^13^C MR signal decay occurs due to the effects of T_1_ as well as the radio frequency (RF) excitation, which follows Equation E1.1 (modified from ^1^):

$M(t)=M_{\infty}e^{-t\left( \frac{-\log\left( \cos\left( c*FA \right) \right)}{TR}+\frac{1}{T_{1}} \right)}+M_{o}$ (E1.1)

where $M_{\infty}$ is the steady state ^13^C magnetization under microwave irradiation, *c* is the calibration factor for flip angle, *FA* is flip angle applied, *TR* is repetition time, and $M_{o}$ is the thermal equilibrium magnetization.

Equation E1.1 can be simplified as follows:

$M(t)=M_{\infty}e^{-tK_{FA}}+M_{o}$ (E1.2)

As $M\left( t \right)\gg M_{o}$, equation E1.2 can be further simplified as:

$M\left( t \right)=M_{\infty}e^{-tK_{FA}}$ (E1.3)

In equations E1.2 and E1.3, *K_FA_* is the exponential decay constant, which depends on the T_1_ as well as the RF excitation at specified flip angle *FA*, as described by Equation E1.4.

$K(FA)=\frac{-\log\left( \cos\left( c*FA \right) \right)}{TR}+\frac{1}{T_{1}}$ (E1.4)

Thus, to determine T_1_, we performed consecutive pulse-acquire experiments, using flip angles (FA) of 5°, 10°, 15°, and 20°, with 5 repetitions for each flip angle, and repetition time of 1 s. A representative plot of decay in the signal amplitude of hyperpolarized [3-^13^C]acetoacetate, upon dissolution, is shown in Figure M1.


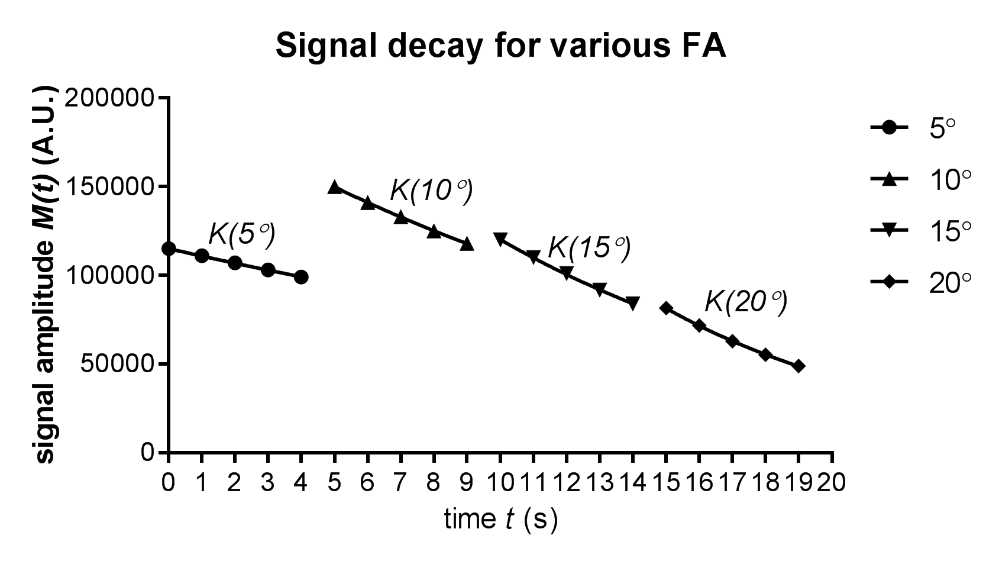


**Figure M1**. Amplitude of hyperpolarized [3-^13^C]acetoacetate following dissolution, during pulse-acquire experiments using various flip angles (FA) and repetition time of 1s.

Exponential decay constant *K_FA_* for each flip angle can then be determined by fitting the data in Figure M1 using Equation E1.3. Data fitting using Equation E1.3 yields *K(5°), K(10°), K(15°),* and *K(20°)*. The representative plot of flip angles against the corresponding exponential decay constants is shown in Figure M2. T_1_ can then be determined by fitting the data in Figure M2 using Equation E1.4.


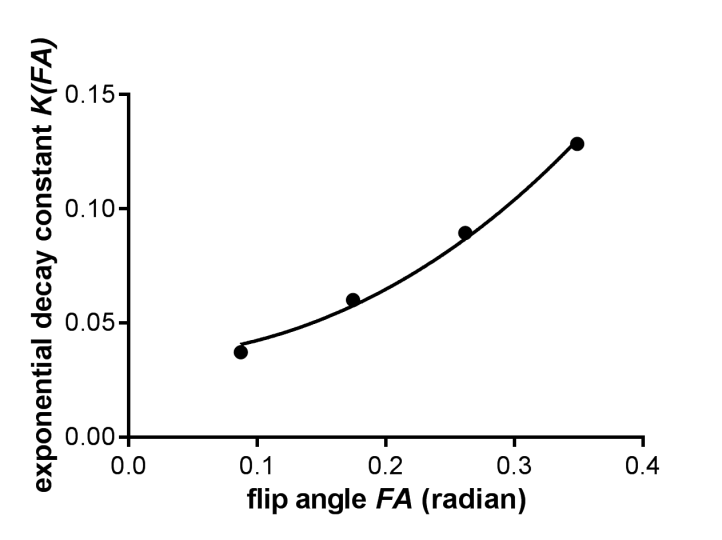


Figure M2. Plot of flip angles vs. the corresponding exponential decay constants.

***Kinetic modelling to calculate metabolic conversion rates from ^13^C MRS dynamic data***

Kinetic modelling was performed using a modification of the formula described by Atherton et al. ^2^ First, the [3-^13^C]acetoacetate, [5-^13^C]glutamate and [1-^13^C]acetylcarnitine peaks at each time point during the acquisition duration were fitted in jMRUI. To improve SNR, the fitted amplitudes were first summed over 3 adjacent time points. The [3-^13^C]acetoacetate data as a function of time $M_{AcAc}\left( t \right)$ were then fitted using Equations E2.1 and E2.2, to fit the rate constant for acetoacetate signal decay $k_{AcAc}$ (s^−1^), acetoacetate arrival rate ${rate}_{inj}$ (A.U. s^−1^), and acetoacetate arrival time $t_{arrival}$ (s). The parameter $t_{end}$ is the sum of $t_{arrival}$ and the injection duration.

$M_{AcAc}\left( t \right)=\frac{{rate}_{inj}}{k_{AcAc}} \left( 1-e^{-k_{AcAc}(t-t_{arrival}} \right) , t_{arrival}\leq t<t_{end}$ (E2.1)

$M_{AcAc}\left( t \right)=M_{AcAc}\left( t_{end} \right)e^{-k_{AcAc} (t-t_{end})} , t\geq t_{end}$ (E2.2)

Using the fit parameters of acetoacetate (i.e.,$k_{AcAc}, {rate}_{inj}$, and $t_{arrival}$), the metabolite data as a function of time $M_{x}\left( t \right)$ were fit using Equation E2.3 and E2.4.

$M_{x}\left( t \right)=\frac{k_{AcAc\to x}{rate}_{inj}}{k_{AcAc}-k_{x}} \left( \frac{1-e^{-k_{x}\left( t-t_{arrival} \right)}}{k_{x}}-\frac{1-e^{-k_{AcAc}\left( t-t_{arrival} \right)}}{k_{AcAc}} \right), t_{arrival}\leq t<t_{end}$ (E2.3)

$$M_{x}\left( t \right)=\frac{M_{AcAc}\left( t_{end} \right)k_{AcAc\to x}}{k_{AcAc}-k_{x}}\left( e^{-k_{x}\left( t-t_{end} \right)}- e^{-k_{AcAc}\left( t-t_{end} \right)} \right)+M_{x}\left( t_{end} \right)e^{-k_{x} \left( t-t_{end} \right)},$$

$t\geq t_{end}$ (E2.4)

In this case, $M_{x}\left( t \right)$ is either [5-^13^C]glutamate or [1-^13^C]acetylcarnitine peak area as a function of time. The equations fit the rate constant for acetoacetate to metabolite exchange and $k_{AcAc\to x}$(s^−1^), and the rate constant for metabolite signal decay $k_{x}$(s^−1^).

**Supplementary Tables**

**Supplementary Table S1.** Calculated acetoacetate concentration in the blood immediately after injection of lithium [3-^13^C]acetoacetate.

|  | Control | GK |
| --- | --- | --- |
| Body weight (g) | 479.8 | 403.3 |
| Total blood volume* (mL) | 30.7 | 25.8 |
| *Prior to injection* |  |  |
| Measured serum AcAc concentration^#^ (mmol/L) | 0.52 | 0.55 |
| Amount of AcAc in blood (mmol) | 0.016 | 0.014 |
| *Injection of 80 mM lithium [3-^13^C]acetoacetate,*  *at a dose of 0.24 mmol/kg* |  |  |
| Amount of AcAc injected (mmol) | 0.115 | 0.097 |
| Total of AcAc in the blood (mmol) | 0.131 | 0.111 |
| Concentration of AcAc in the serum (mmol/L) | 4.27 | 4.30 |

*Total blood volume is estimated from 64 ml/kg. ^#^Serum AcAc concentration at baseline was measured from blood sample collected at sacrifice.

**Supplementary Table S2.** Lithium concentration measured 90 minutes after lithium [3-^13^C]acetoacetate injection.

|  | Serum lithium concentration (mmol/L) |
| --- | --- |
| Controls (n=6) | 0.35 ± 0.09 |
| GK (n=8) | 0.24 ± 0.03 |

Data are means ± SD.

**Supplementary Figures**

**Supplementary Figure S1. ^13^C NMR of [3-^13^C]ethyl acetoacetate.** An impurity peak at ~180 ppm which appears in the ^13^C MR spectrum of dissoluted lithium [3-^13^C]acetoacetate is not present in the ^13^C MR spectrum of [3-^13^C]ethyl acetoacetate.


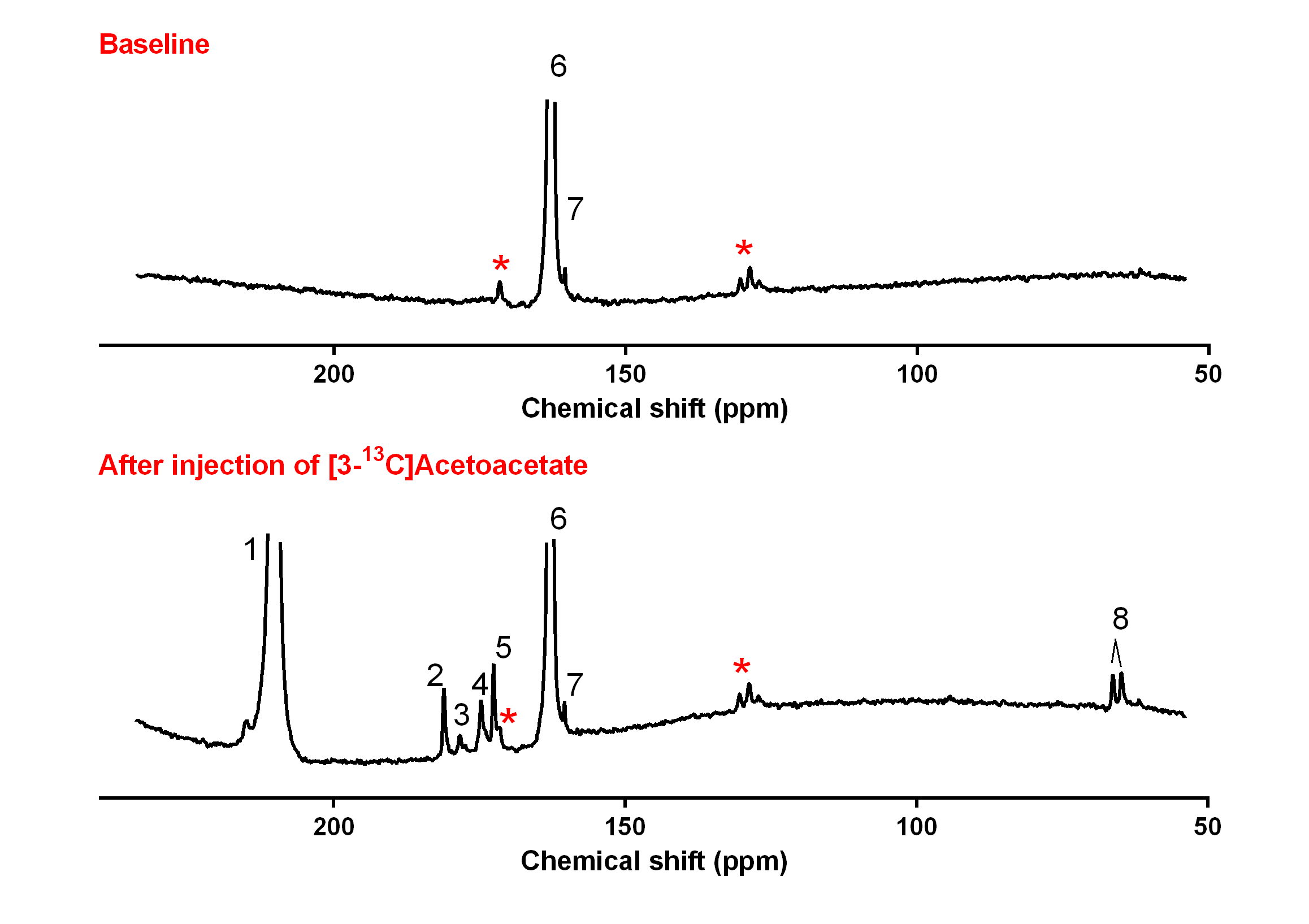


**Supplementary Figure S2. Comparisons of cardiac ^13^C MR spectra acquired at baseline and after injection of hyperpolarized [3-^13^C]acetoacetate**. 1: [3-^13^C]acetoacetate, 2: [5-^13^C]glutamate, 3: [5-^13^C]citrate, 4: [1-^13^C]acetoacetate, 5: [1-^13^C]acetylcarnitine, 6: [1-^13^C]urea, 7: impurity from [1-^13^C]urea, 8: [3-^13^C]β-OHB. Asterisks (*) indicate peaks that appear in both spectra, which originate from endogenous ^13^C, potentially lipids ^3,4^.

**
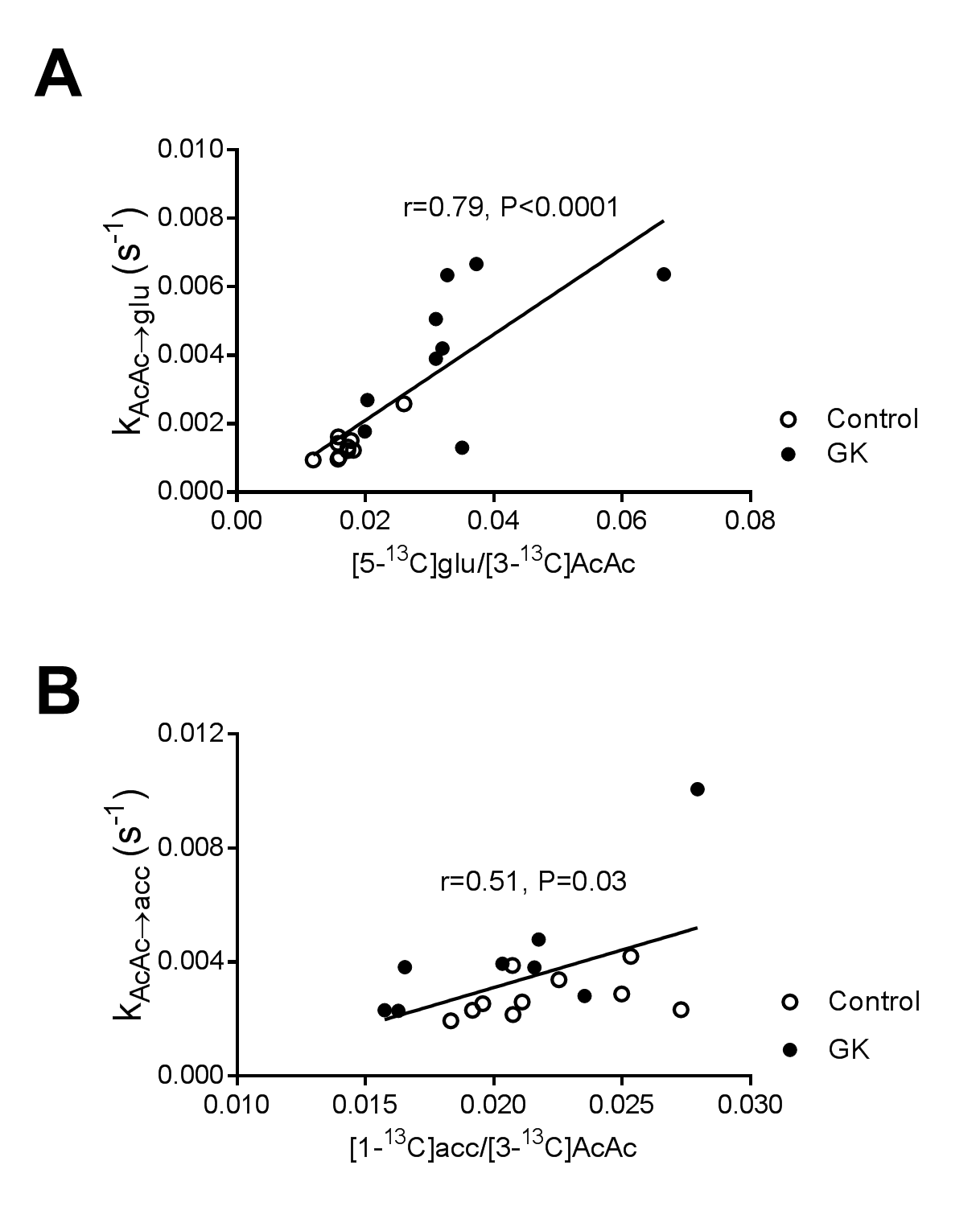
**

**Supplementary Figure S3. Correlations between metabolic ratios and metabolic conversion rates obtained using kinetic modelling.** (A) [5-^13^C]glutamate/[3-^13^C] acetoacetate vs. metabolic conversion rates for [3-^13^C]acetoacetate to [5-^13^C]glutamate exchange $k_{AcAc\to Glu}$, (B) [1-^13^C]acetylcarnitine/[3-^13^C]acetoacetate vs. metabolic conversion rates for [3-^13^C]acetoacetate to [1-^13^C]acetylcarnitine exchange $k_{AcAc\to acc}$. Controls n=10, GK n=9 (except for (B) GK n=8). AcAc: acetoacetate, acc: acetylcarnitine, glu: glutamate.


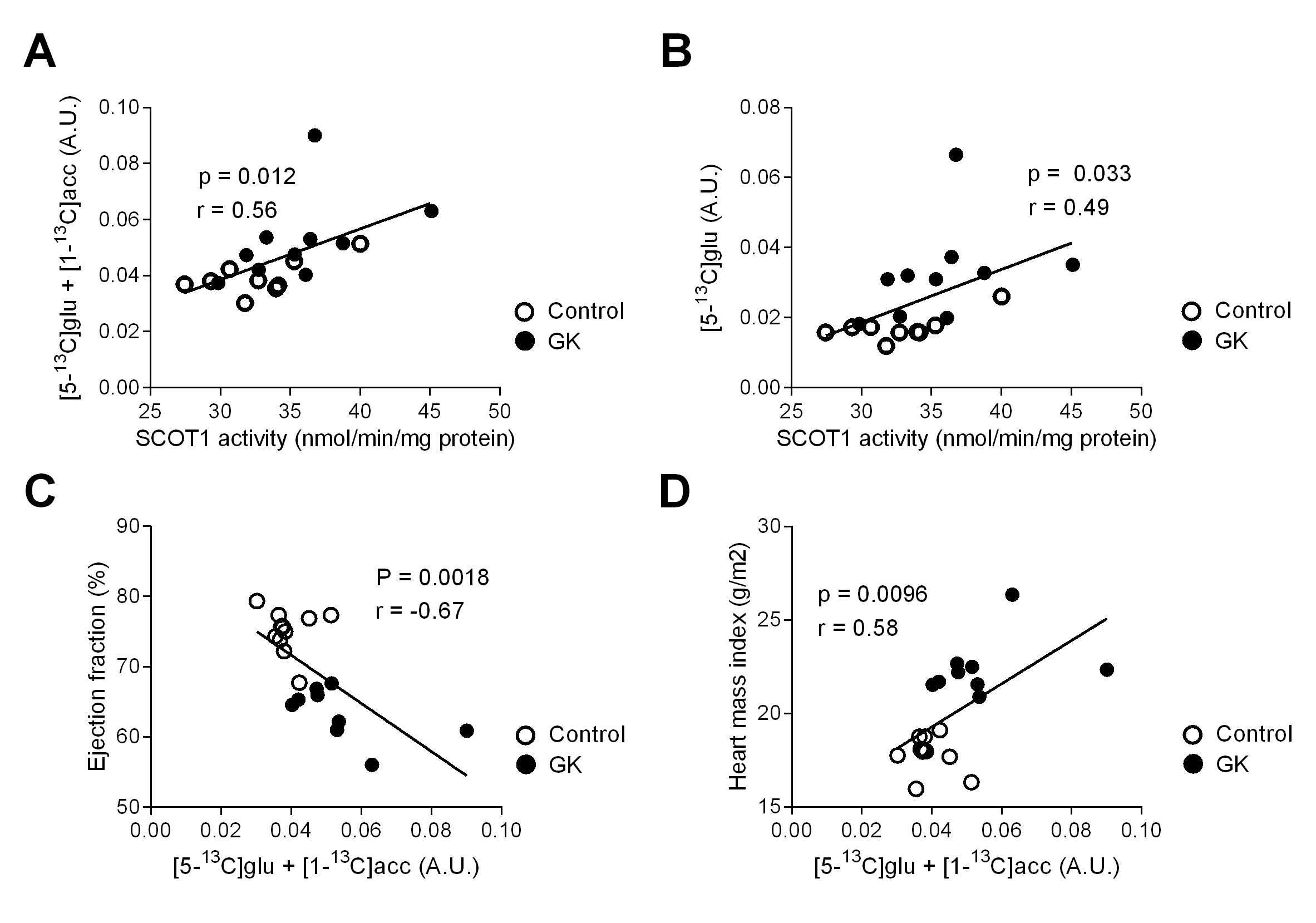


**Supplementary Figure S4. Correlations including all GK data points**. (A) SCOT activity vs. ([5-^13^C]glutamate +[1-^13^C]acetylcarnitine), (B) SCOT activity vs. [5-^13^C]glutamate, (C) ([5-^13^C]glutamate + [1-^13^C]acetylcarnitine) vs. cardiac function, and (D) ([5-^13^C]glutamate + [1-^13^C]acetylcarnitine) vs. heart mass index. Controls n=10, GK n=9. Acc: acetylcarnitine, glu: glutamate.


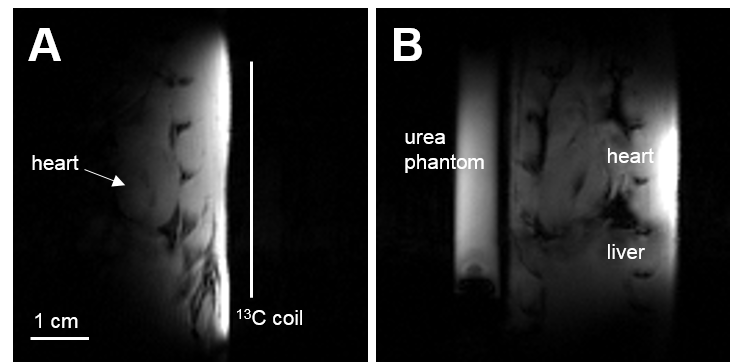


**Supplementary Figure S5. Animal positioning relative to the surface coil.**

(A) Sagittal and (B) coronal scout images (flip angle: 30˚, repetition time: 17 ms, echo time: 2.6 ms, number of averages: 20, matrix size: 128x128, field of view 6x6 cm^2^). Note that the sensitive area for ^1^H is larger than that for ^13^C. The diameter of the ^1^H surface loop coil is 5 cm, while that of the ^13^C surface loop coil is 4 cm.


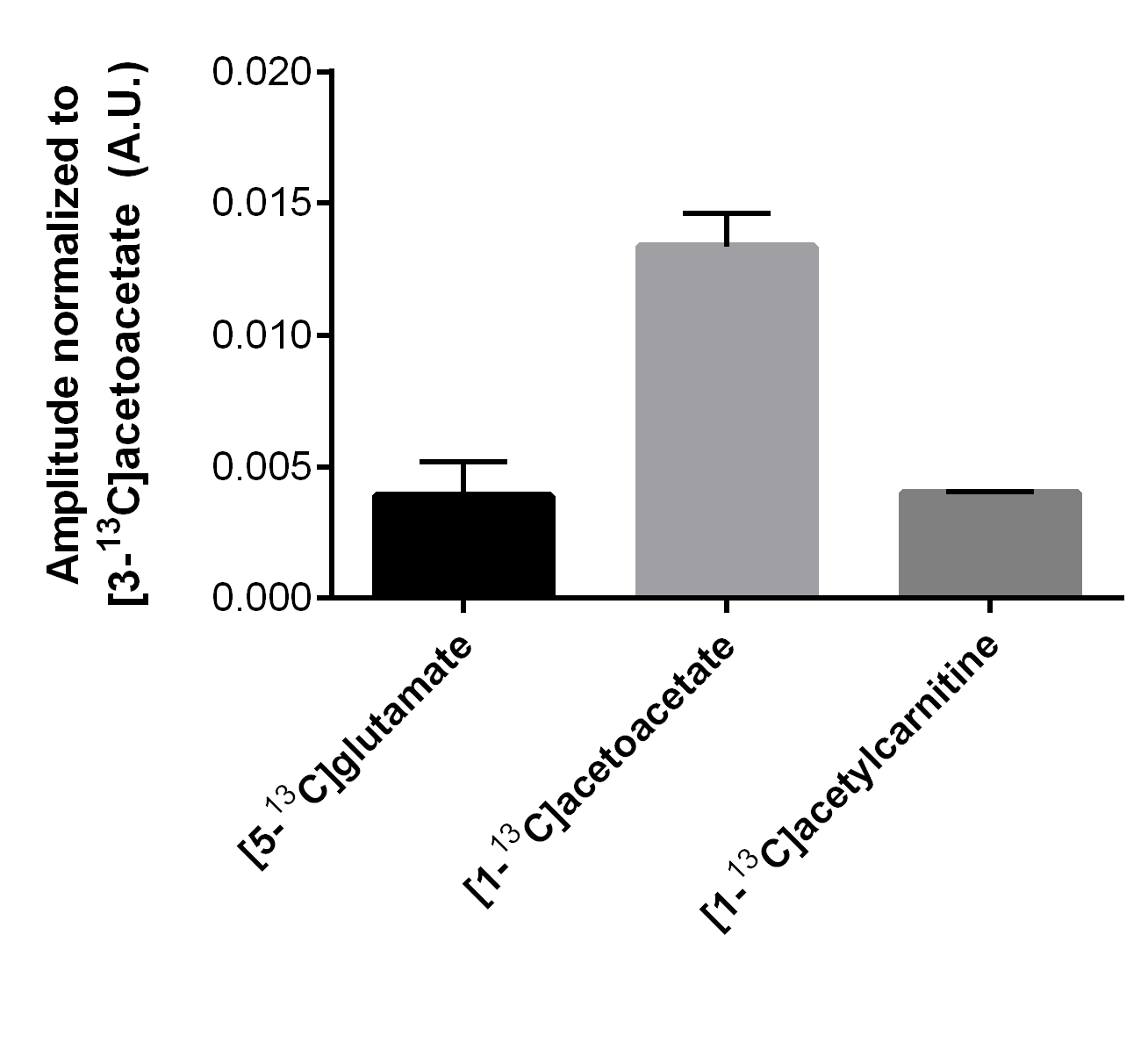


**Supplementary Figure S6. Quantification of metabolic production in the liver upon injection of lithium [3-^13^C]acetoacetate.** Similar to the quantification for cardiac ^13^C MRS data, the quantification was performed on the summed spectra over 30 spectra (60 seconds upon acetoacetate arrival). Data are means ± SD (n=3, except for [1-^13^C]acetylcarnitine n=2 as the [1-^13^C]acetylcarnitine signal was too low for one rat and could not be quantified).

**
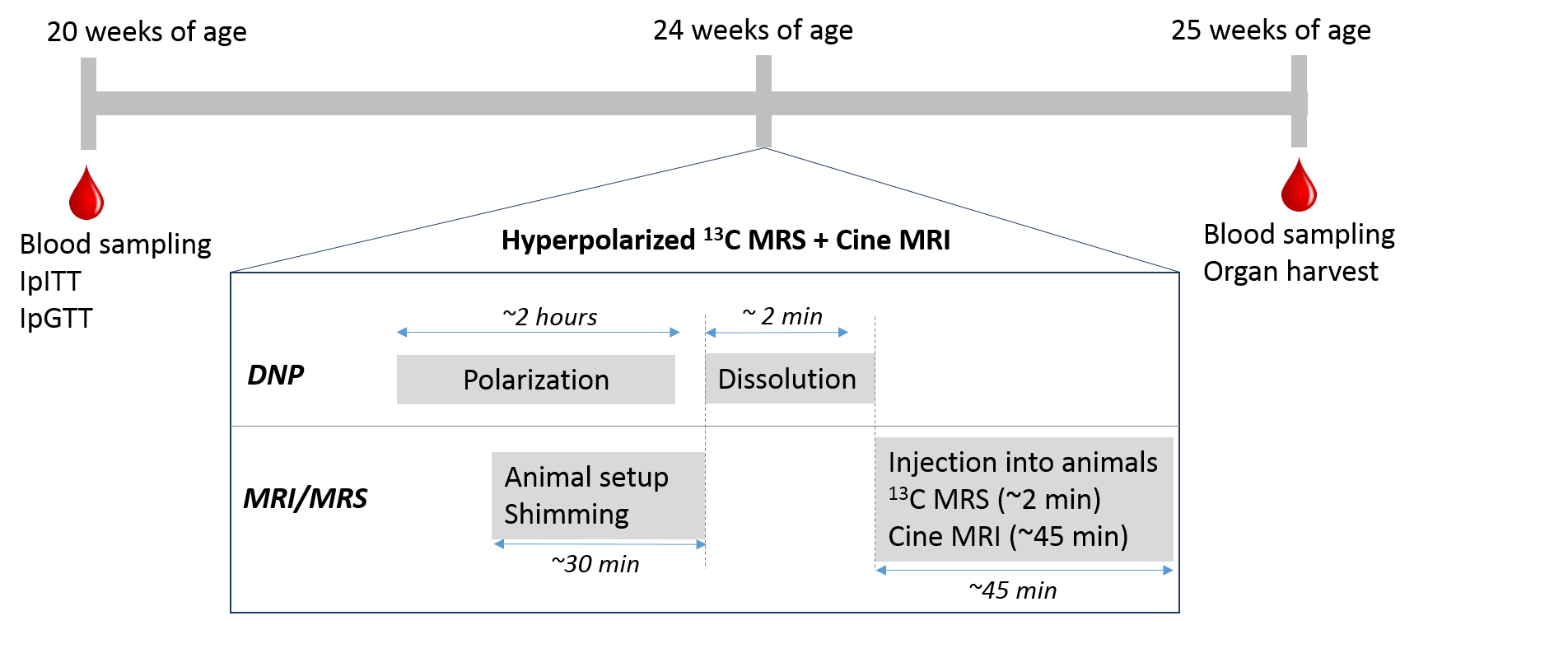
**

**Supplementary Figure S7. Experiment diagram.** Hyperpolarized ^13^C MRS and cine MRI were performed by first polarizing lithium [3-^13^C]acetoacetate for ~2 hours. About 30 minutes before the end of polarization, the animal was set up in the scanner for positioning and shimming. When expected polarization was reached, the hyperpolarized sample was dissoluted then immediately injected into the animal. ^13^C MRS was initiated shortly before injection. Cine MRI was performed in the same session, following ^13^C MRS. MRS: magnetic resonance spectroscopy, MRI: magnetic resonance imaging, DNP: dynamic nuclear polarization.

**References**

1. Filibian, M. *et al.* The role of the glassy dynamics and thermal mixing in the dynamic nuclear polarization and relaxation mechanisms of pyruvic acid. *Phys. Chem. Chem. Phys.* **16,** 27025–27036 (2014).

2. Atherton, H. J. *et al.* Validation of the in vivo assessment of pyruvate dehydrogenase activity using hyperpolarised 13C MRS. *NMR Biomed.* **24,** 201–208 (2011).

3. Soma, M. R., Mims, M. P., Chari, M. V, Rees, D. & Morrisett, J. D. Triglyceride metabolism in 3T3-L1 cells. An in vivo 13C NMR study. *J. Biol. Chem.* **267,** 11168–11175 (1992).

4. Mannina, L. *et al.* Concentration dependence of 13C NMR spectra of triglycerides: implications for the NMR analysis of olive oils. *Magn. Reson. Chem.* **38,** 886–890 (2000).
